# Toward forward genetic screens in malaria-causing parasites using the piggyBac transposon

**DOI:** 10.1186/1741-7007-9-21

**Published:** 2011-03-31

**Authors:** Brendan S Crabb, Tania F de Koning-Ward, Paul R Gilson

**Affiliations:** 1 Macfarlane Burnet Institute for Medical Research and Public Health, Melbourne, Victoria 3004, Australia; 2 The University of Melbourne, Melbourne, Victoria 3010, Australia; 3 Monash University, Clayton, Victoria 3800, Australia; 4 Deakin University, Waurn Ponds, Victoria 3217, Australia

## Abstract

The ability to analyze gene function in malaria-causing *Plasmodium *parasites has received a boost with a recent paper in *BMC Genomics *that describes a genome-wide mutagenesis system in the rodent malaria species *Plasmodium berghei *using the transposon *piggyBac*. This advance holds promise for identifying and validating new targets for intervention against malaria. But further improvements are still needed for the full power of genome-wide molecular genetic screens to be utilized in this organism.

See research article: http://www.biomedcentral.com/1471-2164/12/155

## 

The development of tools for the genetic manipulation of the causative agents of malaria - protozoan parasites of the genus *Plasmodium *- has been arduous and frustratingly slow. The drive to obtain such tools is motivated by the ongoing public-health significance of this disease - malaria remains a global health and economic catastrophe for which new drugs and an effective vaccine are desperately needed, and if these are to be rationally designed, new insights into *Plasmodium *biology, particularly in understanding the function of individual gene products, are in turn required. An illustration of this is provided by the new vaccine candidates that are arising from studies identifying specific parasite antigens as essential or important for red blood cell invasion. Manipulation of the genes encoding these candidates was key to dissecting the relative importance and the precise nature of their biological roles [1]. However, progress in this and related endeavors has been slow, because genetic manipulation of *Plasmodium *parasites remains laborious. Indeed two issues in particular have dogged efforts to develop a array of robust tools to analyze gene function in *Plasmodium*: (1) low transformation efficiency whereby very few parasites in a population receive DNA during an individual transfection experiment, and (2) an inability to use RNA interference approaches (which is an alternative to traditional transformation methods) because the specific RNAi machinery is lacking in this organism [2].

Nevertheless, steady progress is being made and a range of molecular genetic approaches to dissect the function of the 5,300 or so *Plasmodium *genes - including transgenesis, gene knockout, site-directed mutagenesis and knockdown of gene expression - are now available and have been used to good effect [3]. Such tools have been developed both in *Plasmodium falciparum*, the main cause of human malaria, and in the rodent model system *Plasmodium berghei*. In fact the development of tools for investigating *P. berghei *has often outpaced that for *P. falciparum*, largely because of the greater efficiency with which plasmid DNA can be introduced in this organism. Apart from *P. berghei*'s greater tractability to genetic manipulation, this model has much to offer because the entire life cycle - through blood, insect-borne and liver stages - can be readily completed in an experimental setting. This is much more difficult to achieve in any non-rodent system. Because many genes are shared across *Plasmodium *species and are considered orthologous, dissection of gene function in *P. berghei *can be hugely informative to human malaria biology.

Two major obstacles remain in the development of molecular genetic tools for *Plasmodium *species: the lack of a simple but robust system to conditionally ablate the function of genes essential to the blood-stage cycle and the lack of a highly efficient random mutagenesis system to allow classical forward genetic screens to recover mutants with interesting phenotypes. A paper by Fonager *et al*. [4] in *BMC Genomics *has made great strides in addressing the second of these obstacles.

## What's new?

**Figure 1:**
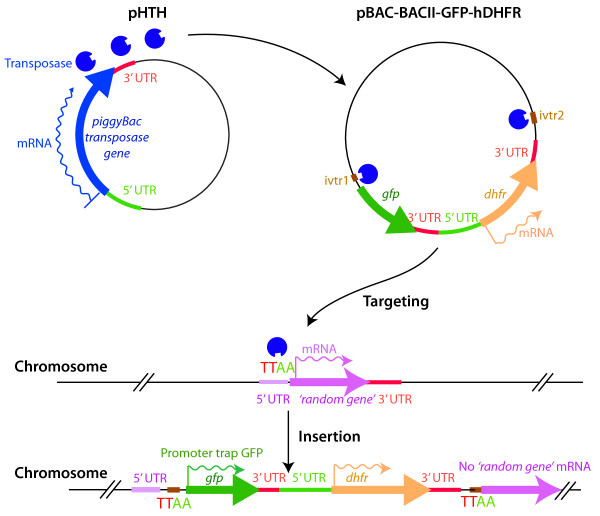
**Use of the *piggyBac *transposon system to disrupt gene function and 'trap' promoter elements**. The *piggyBac *transposase encoded by the pHTH plasmid can integrate a gene cassette (carried on the pBAC-BACII-GFP-hDHFR plasmid) flanked by *piggyBac*-specific inverted repeats into random TTAA sites within the *P. berghei *genome [4]. The gene cassette contains a promoter-less green fluorescent protein (*gfp*) gene and a human dihydrofolate reductase (*hdhfr*) selectable marker. The function of *P. berghei *genes can be ablated by disrupting their coding sequences or their promoters by insertion of the *gfp-hdhfr *cassette. If the cassette inserts downstream of a gene promoter (as shown here) this promoter can drive expression of *gfp*, which provides information about the timing of expression of the disrupted gene.

Fonager and colleagues [4] have developed a system that introduces the *piggyBac *transposon into the genome of *P. berghei*. Originally recognized in virus-infected insect cells, *piggyBac *has been developed as a tool for insertion mutagenesis in a wide range of systems, including mammalian cells. DNA elements flanked by *piggyBac *sequences can insert into TTAA sequences throughout any genome, as long as the cells also express the piggyBac transposase, the enzyme required for *piggyBac *transposition (Figure 1).

Fonager *et al*. [4] build on earlier successes in the Adams laboratory using the same transposon to mutate *P. falciparum *[5,6]. What is new in the current study, apart from the fact that the system has been modified for *P. berghei*, is the markedly improved efficiency of insertion, some 16-18 times that seen in *P. falciparum*. In the *P. berghei *system, 50 or more insertion events can be achieved in a single transfection. Moreover, integration was encouragingly random and included frequent integration into coding sequences, which was not commonly seen in *P. falciparum*. This approach can therefore be used to disrupt gene expression (see Figure 1), and the authors demonstrate targeting by *piggyBac *of genes for proteins non-essential to blood-stage growth [4].

Another advance described is the stable integration of the transposase gene into the genome of *P. berghei*. The consequent continuous expression of the transposase allows excision of an integrated *piggyBac *element and its reinsertion at another genomic location, thus increasing the potential coverage of transposon insertion resulting from a single transfection experiment [4]. Together, these features mean that screening for genes responsible for particular phenotypes is now more feasible and practicable in *Plasmodium *parasites. Demonstrating the potential for such 'forward genetic' screens, Fonager *et al*. [4] show that it is possible to identify transcriptional promoters in the *P. berghei *genome by 'trapping' a promoter-less green fluorescent protein marker gene flanked by *piggyBac *elements downstream of a functional promoter (Figure 1).

## Where to now?

So what is the potential of the *piggyBac *mutagenesis system for genome-wide screens in *P. berghei*? For example, will it be possible to identify at a genome-wide level all the genes essential, or dispensable, for blood-stage growth? To date, several medium-scale gene-knockout approaches have been published, obtaining 20-80 gene-targeting events per study [7,8]. These programs were enormously labor intensive, however, and fall far short of the genome-wide knockouts needed.

Another question to be asked of any new technique is can it identify the gene(s) responsible for a measurable phenotype, the genetic equivalent of finding a needle in haystack. Such forward genetic screens are particularly needed for *Plasmodium *parasites, in which nearly 50% of *Plasmodium *genes have no recognizable orthologs outside the genus, as no previous knowledge about gene function is required [9]. In forward genetic screens in model organisms, genomes are generally randomly mutated by radiation, chemical mutagens or insertional mutagenesis using transposons, after which mutants are identified by phenotypic assay and the phenotype tied back to the gene that has been mutated. Such approaches require mutagenesis at a 'saturation' level where every gene is perturbed in a single experiment. While some progress has been made in achieving insertional mutagenesis in *Plasmodium*, the systems developed to date to not approach the 'saturation' levels required.

It is probably fair to say that even with the improved efficiency of *piggyBac *insertion demonstrated by Fonager *et al*. [4], this system still falls short of a 'saturating' mutagenesis procedure that could be easily used for screens. The authors estimate that up to 50 transfection experiments would need to be performed to achieve this level of integration. Most promise for the future probably lies in further improvements to the re-mobilization approach demonstrated by Fonager *et al*., in which the transposase remains expressed in the parasite cell and facilitates the transposition of *piggyBac *from one insertion site to another. More frequent and controllable re-mobilization is probably required to make such an approach effective in achieving frequent insertion events. Perhaps this could be achieved by higher-level expression of the transposase and by controlling expression of the transposase with a conditionally regulatable system. Several methods for doing this are now emerging.

Because parasites are grown and transfected in the haploid blood-stages, the *piggyBac *insertion approach will, unfortunately, only be useful for the functional analysis of genes that are not essential to blood-stage survival, that is, it will not be possible to recover mutants in which *piggyBac *has inserted into genes that have a role in blood-stage development. The very essentiality of these genes is of particular interest, as they are likely to be the best targets for future drugs and vaccines. To investigate essential genes will require combining the *piggyBac *system with one of the conditional expression systems currently available. One possibility would be to place the anhydrotetracycline (ATc)-regulated gene-expression system within the *piggyBac *inverted terminal repeats [3], allowing the *piggyBac*-flanked ATc-regulated promoter to jump into and replace the promoter of a random gene. The ATc-regulated promoter would be in the switched-on state, in case the downstream random gene is essential. Once parasites containing these inserted promoters have been cloned, the addition of ATc would switch off expression of the targeted gene, producing a rapid and observable phenotype if the gene is essential.

In summary, this work in *P. berghei *[4] builds on the less efficient *piggyBac *mutagenesis system developed for *P. falciparum *and provides a clear path for saturating genome-wide mutagenesis to be achieved in this organism. Such approaches are needed if the huge amount of genomic information now available is to be dissected and exploited to produce new tools for malaria control. This need is as urgent as ever, given the continued lack of a licensed malaria vaccine and concern over the emergence of parasite strains tolerant to artemisinin derivatives [10].
